# Regional disparities in the prevalence and correlated factors of myopia in children and adolescents in Gansu, China

**DOI:** 10.3389/fmed.2024.1375080

**Published:** 2024-08-01

**Authors:** Jinyu Wang, Sheng Li, Shiqi He, Yali Feng, Pu Li

**Affiliations:** ^1^School of Public Health, Lanzhou University, Lanzhou, China; ^2^Department of Public Health, Lanzhou Second People’s Hospital, Lanzhou, China; ^3^Department of Ophthalmology, Baiyin Second People's Hospital, Baiyin, China

**Keywords:** children and adolescents, myopia, influencing factors, regional disparity, prevention and control

## Abstract

**Background:**

Myopia is a significant public health problem across the globe. This study aimed to examine the regional disparity in prevalence and correlated factors of myopia in children and adolescents in two typical regions, Gannan Tibetan Autonomous Prefecture (Gannan Prefecture for short, a Tibetan residential area) and Wuwei City (a Han residential area) in Gansu Province, China, and to provide a reference for the prevention and control of regional myopia.

**Methods:**

The study was a cross-sectional study of children and adolescents in Gansu Province, China. A total of 6,187 (Wuwei City: 3,266, Gannan Tibetan Autonomous Prefecture: 2,921) students were selected by stratified cluster sampling. Eye examinations and questionnaires were administered to the participants. Myopia is defined as a condition in which the spherical equivalent refractive error of an eye is less than or equal to −0.50 D when ocular accommodation is relaxed. The χ^2^ test and multivariate logistic regression analysis were used to analyze the correlated factors of myopia.

**Results:**

The myopia rate of 6,187 students was 71.4%, and students had a higher rate of myopia (77.5%) in Wuwei City compared to Gannan Prefecture (64.6%) (*p* < 0.001). The results of multivariate analysis in Wuwei City showed that girls (odds ratio (*OR*) = 1.325), junior students (*OR* = 2.542), senior students(*OR* = 4.605), distance between eyes and book less than one foot (*OR* = 1.291), and parents with myopia (one, *OR* = 2.437; two, *OR* = 4.453) had higher risks of myopia (all, *p* < 0.05). For Gannan Prefecture, girls (*OR* = 1.477), senior students (*OR* = 1.537), daily time spent doing homework ≥2 h (OR = 1.420), the distance between eyes and book less than one foot (*OR* = 1.205), mean time continuous eye use (0.25–<0.5 h, *OR* = 1.345, 0.5–<1 h, *OR* = 1.317, ≥1 h, *OR* = 1.313), average daily sleep duration <8 h (*OR* = 1.399), and parents with myopia (one, *OR* = 1.852; two, *OR* = 2.913) had higher risks of myopia (all, *p* < 0.05).

**Conclusion:**

The prevalence of myopia is at a relatively high level in Gansu Province. The prevalence and risk factors for myopia vary by region.

## 1 Introduction

Myopia is currently widely recognized as a significant public health issue causing significant visual loss and a risk factor for a range of other serious ocular conditions (1). Myopia can increase the risk of eye complications such as myopic macular degeneration, retinal detachment, and open-angle glaucoma (2). Myopia also imposes a heavy economic burden. It is estimated that the global productivity loss due to uncorrected refractive errors amounts to $202 billion per year (3).

Myopia is a significant public health problem across the globe. In recent years, there has been a significant increase in the number of cases of myopia globally. It was estimated that 22.9% of the world’s population had myopia in 2020, and it is projected to double in prevalence by the year 2050 (4). Myopia has become a health problem affecting children and adolescents. The prevalence of myopia has been shown to vary widely with geographic location. For example, in Germany from 2014 to 2017, the prevalence of myopia among children and adolescents 0 to 17 years of age was 11.4% (5). Myopia was found in 3.7% of children aged 6–7 years old and 22.8% of children aged 12–13 years old in Ireland (6). However, the level of myopia in Asian countries is considerably higher in East and Southeast Asia. For example, the prevalence rates of myopia were 76.5% among elementary school students and 94.9% among junior high school students (7), 64.6% of children aged 5–18 years old in Korea (8), and 81.2% of children aged 16–18 years old in China (9). Multiple measures and much effort have been taken to curb the prevalence of myopia.

One important measure to enable the effective prevention of myopia is understanding the modifiable risk factors. The high prevalence of myopia is associated with a variety of factors. Previous studies indicated that a family history of myopia is important in myopia occurrences, with an estimated adjusted OR of 2.83 [95% confidence interval (95%CI), 2.47–3.24] in children with two myopic parents compared to no parental myopia in China (10); especially for high myopia, the additive genetic portion remained roughly constant at approximately 60% in South Korea (11). Environmental factors also play a role in the etiology of myopia (12). For example, associations between time outdoors and less myopia were stronger and more consistently observed; daily outdoor time of 120 to 150 min at 5,000 lux/min or cumulative outdoor light intensity of 600,000 to 750,000 lux significantly reduced the incidence risk ratio of myopia by 15% ~ 24% in Shanghai, China (13, 14).

A previous study showed that myopia displayed a regionalization feature, and the incidence of myopia in Gansu Province was at the middle level in China (15). Gansu Province, located in northwest China, is a multi-ethnic populated area. The population of Gansu Province is mainly Han, in addition, there are also 53 other ethnic groups, such as Tibetans. Wang et al. (16) found that prevalence and risk factors for myopia varied between Han and Yugur older adults in Gansu Province. Therefore, we speculate whether there are different factors affecting myopia among primary and secondary school students in different ethnic residential areas in Gansu Province. Based on this, Gannan Tibetan Autonomous Prefecture (Gannan Prefecture for short, a Tibetan residential area) and Wuwei City (a Han residential area) were selected. The purpose of the present study was to examine the variability of prevalence and associated risk factors of myopia in children and adolescents from the Han and Tibetan residential regions. Identification of modifiable correlative factors of myopia in different population settings may help provide a reference for the regional eye health intervention.

## 2 Materials and methods

### 2.1 Participants

This is a cross-sectional survey in Gansu Province. Gansu Province is located in the west of China. There are 55 ethnic groups in Gansu Province, with the Han being the main ethnic group. Tibetans are one of the most populous ethnic minorities. In this study, we selected two typical survey areas: (1) Gannan Prefecture and (2) Wuwei City. Gannan Prefecture is located in the southwest of Gansu Province, where the ethnic composition is mainly Tibetan, Han, Hui, and Tu, and the Tibetan population accounts for more than half of Gannan Prefecture. Wuwei City is located in the central of Gansu Province, with a majority Han population an epidemiological investigation was conducted in the two survey areas during 2020. The stratified cluster sampling method was adopted. First, schools were stratified by urban/rural status, and by elementary/junior/senior status, where we randomly selected 22 schools, including 6 senior high schools, 8 junior high schools, and 8 elementary schools from these two regions. Second, a total of 6,271 students from grades four to six in elementary school, grades seven to nine in junior high schools, and grades one to three in senior high schools were selected as a whole class from the 22 schools. These 6,271 students underwent myopia screening and questionnaire survey. We excluded students with eye diseases other than refractive defects or those who underwent eye surgery. After exclusion, 6,187 (98.7%) students were selected to participate in the study: 3,266 from Wuwei City and 2,921 from Gannan Prefecture. This study was approved by the Ethics Committee of the School of Basic Medical Sciences, Lanzhou University (No: jcyxy20200325), with the consent of students and their guardians, and all information was strictly confidential.

### 2.2 Eye examinations and determination of myopia

Eye examinations of all participants by qualified optometrists from Baiyin Second People’s Hospital. First, a 5-meter standard logarithmic visual acuity chart is used for the examination of distant vision. The right eye’s visual acuity was first measured, followed by the left eye. Second, refractive error was carried out to all participants. The international consensus for defining myopia in epidemiological studies suggests that myopia is defined as a condition in which the spherical equivalent refractive error (SE) of an eye is ≤ −0.50 D when ocular accommodation is relaxed (1). Low myopia is defined as a condition in which the SE of an eye is ≤ −0.50 D and > −6.00 D when ocular accommodation is relaxed (1). High myopia is defined as a condition in which the SE of an eye is ≤ −6.00 D when ocular accommodation is relaxed (1). Pre-myopia is defined as a condition in which the SE of an eye is ≤ +0.75 D and > −0.50 D in children where a combination of baseline refraction, age, and other quantifiable risk factors provide a sufficient likelihood of the future development of myopia to merit preventative interventions (1). However, there is evidence of a high correlation between non-cycloplegic and cycloplegic refraction tests in myopia screening, and The China National Health Commission recommends the use of non-cycloplegic for vision screening in schools (17, 18). Therefore, refractive error was measured using an automatic refractometer (VS100, Welch Allyn, Jiangsu, China) when ocular accommodation was non-relaxation. Before the refractive examination, the instrument was placed in a stable environment. During the refractive examination, participants were asked to focus on the vision screen until an aperture appeared on the gray screen. After the inspection, the result was automatically output by the instrument. SE is the sum of the spherical value plus half the cylindrical value.

### 2.3 Questionnaire

We designed a self-administered questionnaire based on “the work plan of monitoring and intervention of common diseases and health effects among students in 2020 in Gansu Province,” normative guidelines, and similar previous studies (17, 19, 20). The questionnaire included three parts: (1) Basic information: residential district, name of school, age, gender (boy, girl), ethnicity (Han, Tibetan, other ethnic), education level (elementary, junior, senior), daytime outdoor activity hours, average daily sleep duration, and parental history of myopia (neither, one, two); (2) ocular behaviors and environment: frequency of daily eye exercises (zero, one time, two, or more times), recess activity space (inside the teaching building, outdoor), daily time spent doing homework, distance between eyes and book more than one feet (never, occasionally, often, always), teachers emphasizing proper reading and writing posture (never, occasionally, often, always), reading while reclined or lying down (never, occasionally, often, always), daily time spent using computer, mean time between continuous eye use (less than 15 min, 15 min–<30 min, 30 min–< 60 min, 60 min, or more), and previous diagnosis or treatment of eye diseases other than refractive defects. The questionnaires were administered to the students and their parents or legal guardians by trained investigators from September to November 2020. Before filling in the questionnaire, the investigators met with the students, their parents, or legal guardians, and explained what the questionnaires were about and how to fill out the questionnaire. The students and their parents or legal guardians discussed the study and gave their consent.

### 2.4 Statistical analysis

The data were statistically analyzed by SPSS 18.0 software. The χ^2^ test was used to compare the difference in demographic characteristics and the prevalence of myopia stratified by region. Variables, which have a *p*-value of less than 0.05 in the univariate analysis of myopia, were selected and evaluated by multivariate logistic regression models. The results of multivariate logistic regression are presented with *ORs* and 95%*CIs*. Every statistical test was two-sided, and a *p*-value of less than 0.05 was regarded as statistically significant.

## 3 Results

### 3.1 Characteristics of study participants

The demographic characteristics stratified by region are described in Table 1. Statistically significant differences between regional groups were observed in the gender, ethnicity, education level, frequency of daily eye exercises, daily time spent doing homework, the distance between eyes and book, teachers emphasizing proper reading, writing posture, reading while reclined or lying down, daily time spent using a computer, mean time continuous eye use, average daily sleep duration, daytime outdoor activity hours, and parental history of myopia (all, *p* < 0.05).

**Table 1:** Demographic characteristics stratified by region.

Variables	Total (*n* = 6,187)	Wuwei city (*n* = 3,266)	Gannan prefecture (*n* = 2,921)	*χ^2^*	*p*
Gender				20.879	<0.001
Boy	3,242 (52.4)	1,801 (55.1)	1,441 (49.3)		
Girl	2,945 (47.6)	1,465 (44.9)	1,480 (50.7)		
Ethnicity				2058.843	<0.001
Ethnic Han	4,569 (73.8)	3,193 (97.8)	1,376 (47.1)		
Tibetan	1,320 (21.3)	35 (1.1)	1,285 (44.0)		
Other Ethnic	298 (4.9)	38 (1.1)	260 (8.9)		
Education level				7.029	0.030
Elementary	2,363 (38.2)	1,218 (37.3)	1,145 (39.2)		
Junior	2,256 (36.5)	1,241 (38.0)	1,015 (34.7)		
Senior	1,568 (25.3)	807 (24.7)	761 (26.1)		
Frequency of daily eye exercises				1206.186	<0.001
0	1,072 (17.3)	268 (8.2)	804 (27.5)		
1	2,653 (42.9)	1,049 (32.1)	1,604 (54.9)		
≥ 2	2,462 (39.8)	1,949 (59.7)	513 (17.6)		
Recess activity space				0.056	0.812
Inside the teaching building	3,430 (55.4)	1,806 (55.3)	1,624 (55.6)		
Outdoor	2,757 (44.6)	1,460 (44.7)	1,297 (44.4)		
Daily time spent doing homework				34.884	<0.001
< 2 h	3,290 (53.2)	1,261 (49.6)	1,669 (57.1)		
≥ 2 h	2,897 (46.8)	1,645 (50.4)	1,252 (42.9)		
Distance between eyes and book				291.357	<0.001
Less than one foot	2,951 (47.7)	1,223 (37.4)	1,728 (59.2)		
More than one foot	3,236 (52.3)	2,043 (62.6)	1,193 (40.8)		
Teachers emphasizing proper reading, writing posture				101.473	<0.001
Occasionally	1,893 (30.6)	817 (25.0)	1,076 (36.8)		
Always	4,294 (69.4)	2,449 (75.0)	1,845 (63.2)		
Reading while reclined or lying down				13.171	<0.001
Occasionally	5,382 (87.0)	2,889 (88.5)	2,493 (85.3)		
Always	805 (13.0)	377 (11.5)	428 (14.7)		
Daily time spent using computer				19.020	<0.001
< 1 h	5,596 (90.4)	3,004 (92.0)	2,592 (88.7)		
1–2 h	429 (7.0)	193 (5.9)	236 (8.1)		
≥ 2 h	162 (2.6)	69 (2.1)	93 (3.2)		
Mean time continuous eye use				116.638	<0.001
< 0.25 h	1,500 (24.2)	622 (19.1)	878 (30.1)		
0.25–<0.5 h	1,279 (20.7)	755 (23.1)	524 (17.9)		
0.5- < 1 h	1,541 (24.9)	875 (26.8)	666 (22.8)		
≥ 1 h	1,867 (30.2)	1,014 (31.0)	853 (29.2)		
Average daily sleep duration				134.193	<0.001
< 8 h	2,587 (41.8)	1,590 (48.7)	997 (34.1)		
≥ 8 h	3,600 (58.2)	1,676 (51.3)	1,924 (65.9)		
Daytime outdoor activity hours				118.233	<0.001
< 1 h	1,732 (28.0)	740 (22.7)	992 (34.0)		
1- < 2 h	1,913 (30.9)	1,010 (30.9)	903 (30.9)		
≥ 2 h	2,542 (41.1)	1,516 (46.4)	1,026 (35.1)		
Parents with myopia				533.250	<0.001
Neither	3,943 (63.7)	1,651 (50.6)	2,292 (78.5)		
One	1,628 (26.3)	1,133 (34.7)	495 (16.9)		
Two	616 (10.0)	482 (14.7)	134 (4.6)		

### 3.2 Univariate analysis of myopia

The prevalence rate of myopia was 71.4% (4,420/6187) for all participants. The univariate analysis of myopia for all participants is shown in Table 2. Statistically significant differences were observed in factors such as region, gender, ethnicity, education level, recess activity space, daily time spent doing homework, the distance between eyes and book, teachers emphasizing proper reading, writing posture, reading while reclined or lying down, mean time continuous eye use, average daily sleep duration, daytime outdoor activity hours, and parental history of myopia (all, *p* < 0.05).

**Table 2:** Univariate analysis of myopia in the whole subjects.

Variables	Myopia numbers	Myopia incidence (%)	*χ^2^*	*p*
Region			125.57	<0.001
Wuwei city	2,532	77.5		
Gannan prefecture	1,888	64.6		
Gender			35.075	<0.001
Boy	2,211	68.2		
Girl	2,209	75.0		
Ethnicity			41.203	<0.001
Ethnic Han	3,363	73.6		
Tibetan	855	64.8		
Other Ethnic	202	67.8		
Education level			246.359	<0.001
Elementary	1,465	62.0		
Junior	1,621	71.9		
Senior	1,334	85.1		
Frequency of daily eye exercises			13.503	0.001
0	767	71.5		
1	1,954	73.7		
≥ 2	1,699	69.0		
Recess activity space			23.498	<0.001
Inside the teaching building	2,536	73.9		
Outdoor	1884	68.3		
Daily time spent doing homework			144.858	<0.001
< 2 h	2,137	65.0		
≥ 2 h	2,283	78.8		
Distance between eyes and book			21.771	<0.001
Less than one foot	2,191	74.2		
More than one foot	2,229	68.9		
Teachers emphasizing proper reading, writing posture			18.091	<0.001
Occasionally	1,422	75.1		
Always	2,998	69.8		
Reading while reclined or lying down			15.400	<0.001
Occasionally	3,798	70.6		
Always	622	77.3		
Daily time spent using computer			0.899	0.638
< 1 h	3,990	71.3		
1–2 h	315	73.4		
≥ 2 h	115	71.0		
Mean time continuous eye use			86.407	<0.001
< 0.25 h	955	63.7		
0.25–<0.5 h	886	69.3		
0.5- < 1 h	1,160	75.3		
≥ 1 h	1,419	76.0		
Average daily sleep duration			260.482	<0.001
< 8 h	2,131	82.4		
≥ 8 h	2,289	63.6		
Daytime outdoor activity hours			11.695	0.003
< 1 h	1,241	71.7		
1- < 2 h	1,416	74.0		
≥ 2 h	1763	69.4		
Parents with myopia			272.931	<0.001
Neither	2,541	64.4		
One	1,330	81.7		
Two	549	89.1		

Univariate analysis of myopia stratified by region is shown in Table 3. In the two regions, statistically significant differences were all observed in factors such as gender, education level, frequency of daily eye exercises, recess activity space, daily time spent doing homework, distance between eyes and book, mean time continuous eye use, average daily sleep duration, and parental history of myopia (all, *p* < 0.05). However, factors such as teachers emphasizing proper reading, writing posture, reading while reclined or lying down, daily time spent using a computer, and daytime outdoor activity hours were associated with myopia only in Wuwei City (all, *p* < 0.05).

**Table 3:** Myopia rate stratified by region (%).

Variables	Wuwei city	*χ^2^*value	*p*-value	Gannan prefecture	*χ^2^*value	*p*-value
Gender		12.085	0.001		34.062	<0.001
Boy	75.2			59.4		
Girl	80.3			69.7		
Ethnicity		3.150	0.207		1.483	0.476
Ethnic Han	77.6			64.2		
Tibetan	80.0			64.4		
Other Ethnic	65.8			68.1		
Education level		310.865	<0.001		83.935	<0.001
Elementary	61.3			62.7		
Junior	84.0			57.0		
Senior	92.1			77.7		
Frequency of daily eye exercises		110.564	<0.001		4.824	0.090
0	89.9			65.4		
1	86.0			65.6		
≥2	71.3			60.4		
Recess activity space		8.160	0.004		16.608	<0.001
Inside the teaching building	79.4			67.9		
Outdoor	75.2			60.6		
Daily time spent doing homework		84.592	<0.001		48.185	<0.001
<2 h	70.8			59.3		
≥2 h	84.2			71.7		
Distance between eyes and book		57.909	<0.001		8.998	0.003
Less than one foot	84.7			66.8		
More than one foot	73.2			61.4		
Teachers emphasizing proper reading, writing posture		48.046	<0.001		2.982	0.084
Occasionally	86.3			66.6		
Always	74.6			63.5		
Reading while reclined or lying down		27.162	<0.001		2.138	0.144
Occasionally	76.2			64.1		
Always	88.1			67.8		
Daily time spent using computer		3.984	0.046		0.063	0.969
<1 h	77.0			64.7		
1- < 2 h	83.9			64.8		
≥2 h	81.2			63.4		
Mean time continuous eye use		55.450	<0.001		21.045	<0.001
<0.25 h	70.9			58.5		
0.25–<0.5 h	71.4			66.2		
0.5- < 1 h	81.7			66.8		
≥1 h	82.5			68.2		
Average daily sleep duration		199.330	<0.001		48.796	<0.001
<8 h	88.1			73.2		
≥8 h	67.5			60.2		
Daytime outdoor activity hours		34.143	<0.001		1.252	0.535
<1 h	82.0			63.9		
1- < 2 h	81.1			66.1		
≥2 h	73.0			64.0		
Parents with myopia		151.633	<0.001		55.744	<0.001
Neither	68.9			61.3		
One	84.5			75.4		
Two	90.9			82.8		

**Figure 1:**
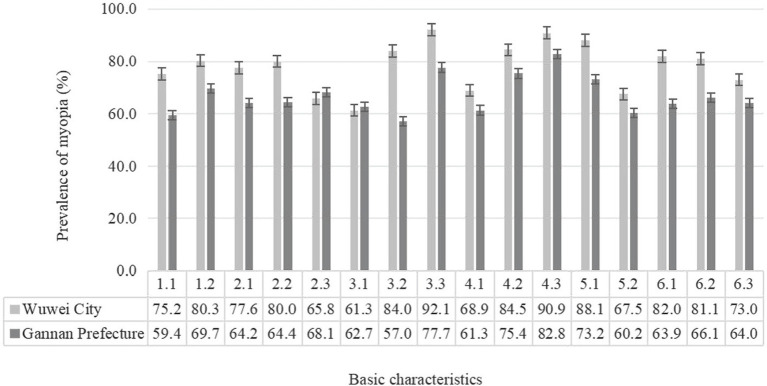
Prevalence of myopia of different basic characters students in the two regions (%). 1.1, Boys; 1.2, girls; 2.1, ethnic han; 2.2, Tibetan; 2.3, other ethnic; 3.1, elementary; 3.2, junior; 3.3, senior; 4.1, no parental myopia; 4.2, father or mother with myopia; 4.3, parents with myopia; 5.1, average daily sleep duration < 8 h; 5.2, average daily sleep duration ≥ 8 h; 6.1, daytime outdoor activity < 1; 6.2, daytime outdoor activity 1–2 h; 6.3, daytime outdoor activity ≥2 h.

**Figure 2:**
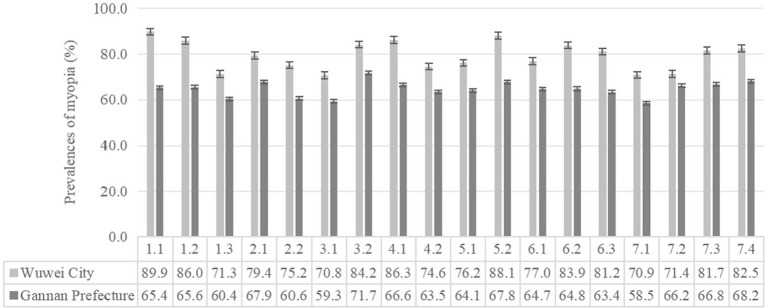
Prevalence of myopia of different ocular behaviors students in the two regions (%). 1.1, doing eye exercises 0 time/d; 1.2, doing eye exercises 1 time/d; 1.3, doing eye exercises ≥2 times/d; 2.1, recess in the teaching building; 2.2, recess outdoor; 3.1, daily time spend doing homework < 2 h; 3.2, daily time spend doing homework ≥2 h; 4.1, distance between eyes book less than one foot; 4.2, distance between eyes book more than one foot; 5.1, reading occasionally while reclined or lying down; 5.2, reading always while reclined or lying down; 6.1, daily time spend using computer < 1 h; 6.2, daily time spend using computer 1–2 h; 6.3, daily time spend using computer ≥2 h; 7.1, mean time continuous eye use < 0.25 h; 7.2, mean time continuous eye use 0.25–0.5 h; 7.3, mean time continuous eye use 0.5–1 h; 7.4, mean time continuous eye use ≥1 h.

Figures 1, 2 show the prevalence of myopia of different basic characters and different ocular behaviors of students in the two regions, respectively. The results showed that the myopia rates were higher in Wuwei City than those in Gannan Prefecture, and there were statistical differences in myopia rates of other groups between the two regions except primary school students and other ethnic students.

### 3.3 Multivariate analysis of myopia

With myopia as a dependent variable (0, no, 1, yes), factors with statistical significance in the results of the univariate analysis were included as covariates in the multivariate logistic regression model for analysis. The backward deletion method is used to screen covariates, and *α* = 0.10 is the criterion for removing covariates. The covariables and assignments in logistic regression analysis are shown in Table 4. After adjustment of confounding variables that were significant in the corresponding univariate analysis, the results of multivariate analysis are shown in Table 5.

**Table 4:** Covariates and assignments of logistic regression analysis.

Variables	Assignments
Region	1: Gannan Prefecture; 2: Wuwei City
Gender	1: Boy; 2: Girl
Ethnicity	1: Ethnic Han; 2: Tibetan; 3: Other Ethnic
Education level	1: Elementary; 2: Junior; 3: Senior
Frequency of daily eye exercises	1: 0 time/d; 2: 1 time/d; 3: ≥2 times/d
Recess activity space	1: Inside the teaching building; 2: outdoor
Daily time spent doing homework	1: <2 h/d; 2: ≥2 h/d
Distance between eyes and book	1: More than one foot; 2: less than one foot
Teachers emphasizing proper reading, writing posture	1: Always; 2: Occasionally
Reading while reclined or lying down	1: Always; 2: Occasionally
Daily time spent using computer	1: <1 h/d; 2: 1- < 2 h/d; 3: ≥2 h/d
Mean time continuous eye use	1: <0.25 h; 2: 0.25–<0.5 h; 3: 0.5- < 1 h; 4: ≥1 h
Average daily sleep duration	1: ≥8 h/d; 2: <8 h/d
Daytime outdoor activity hours	1: <1 h/d; 2: 1- < 2 h; 3: ≥2 h
Parents with myopia	1: Neither; 2: One; 3: Two

**Table 5:** Results of multivariate logistic regression analysis.

Variables	Total		Wuwei city		Gannan prefecture	
	OR (95%CI)	*p*	OR (95%CI)	*p*	OR (95%CI)	*p*
Gender						
Girl	1.393 (1.238, 1.569)	<0.001	1.321 (1.102, 1.583)	0.003	1.480 (1.263, 1.735)	<0.001
Education						
Elementary	Ref		Ref		Ref	
Junior	1.212 (1.043, 1.408)	0.012	2.487 (1.930, 3.204)	<0.001	0.749 (0.616, 0.910)	0.004
Senior	2.183 (1.750, 2.723)	<0.001	4.393 (2.971, 6.496)	<0.001	1.542 (1.168, 2.037)	0.002
Frequency of daily eye exercises						
0	Ref		Ref		Ref	
1	1.060 (0.866, 1.298)	0.570	0.657 (0.417, 1.038)	0.072	0.943 (0.732, 1.214)	0.648
≥2	0.714 (0.598, 0.852)	<0.001	0.576 (0.372, 0.893)	0.014	0.741 (0.600, 0.915)	0.005
Daily time spent doing homework						
≥2 h	1.389 (1.222, 1.578)	<0.001	1.191 (0.974, 1.457)	0.089	1.424 (1.200, 1.689)	<0.001
Distance between eyes and book						
Less than one foot	1.257 (1.108, 1.425)	<0.001	1.278 (1.041, 1.569)	0.019	1.204 (1.024, 1.416)	0.025
Mean time continuous eye use						
<0.25 h	Ref		Ref		Ref	
0.25–<0.5 h	1.213 (1.024, 1.438)	0.025	1.080 (0.835, 1.397)	0.556	1.346 (1.065, 1.701)	0.013
0.5- < 1 h	1.333 (1.125, 1.579)	0.001	1.186 (0.903, 1.557)	0.220	1.319 (1.057, 1.645)	0.014
≥1 h	1.233 (1.046, 1.453)	0.013	1.055 (0.805, 1.383)	0.696	1.307 (1.059, 1.613)	0.013
Average daily sleep duration						
<8 h	1.415 (1.199, 1.669)	<0.001	1.198 (0.921, 1.558)	0.179	1.408 (1.136, 1.746)	0.002
Parents with myopia						
Neither	Ref		Ref		Ref	
One	2.194 (1.889, 2.549)	<0.001	2.443 (1.996, 2.989)	<0.001	1.854 (1.476, 2.328)	<0.001
Two	3.822 (2.916, 5.009)	<0.001	4.352 (3.104, 6.102)	<0.001	2.950 (1.853, 4.698)	<0.001
Region						
Wuwei city	1.813 (1.572, 2.090)	<0.001				

For the whole participants, compared to their respective references, Wuwei City students, girls, junior and senior students, daily time spent doing homework ≥2 h, the distance between eyes and book less than one foot, mean time continuous eye use ≥0.25 h, average daily sleep duration <8 h, and parents with myopia had higher risks of myopia. However, doing daily eye exercises two or more times had a lower risk of myopia (all, *p* < 0.05).

For the participants from Gannan Prefecture, the influencing factors of myopia were consistent with the whole group. For the participants in Wuwei City, the factors affecting myopia only included girls, junior students, senior students, distance between eyes and books less than one foot, and parents with myopia doing daily eye exercises two or more times (all, *p* < 0.05).

## 4 Discussion

This study investigated myopia status and its correlated factors in children and adolescents in two typical regions in Gansu Province, China. Our results suggested that there was a relatively high prevalence of myopia in Gansu. The multivariate analysis showed some factors, which included gender, education, heredity, and environmental factors, correlated with myopia. When stratified by region, the results also showed the regional disparity in prevalence and correlated factors of myopia.

According to the current study, the prevalence of myopia in Gansu (71.4%) was higher than the levels of Asia (60%) and North America (42%) (21), and the results also displayed the regional disparity in prevalence of myopia. Myopia has become an important health problem for children and adolescents in Gansu. Identifying the factors related to myopia, which vary by region, is crucial for reducing its incidence and promoting population health.

Previous studies showed that heredity might play an important role in myopia (22). Rathi et al. (23) reported that the proportion was 30.1, 19.3%, and 13.% among children with myopia in two parents, mother and father, in North India, respectively. Lim et al. (10) reported an *OR* (2.83) in children with two myopic parents compared to no parental myopia. The current study also showed the importance of parental myopia for the myopia of students. For the whole participants, a father or mother with myopia (*OR* = 2.194) and parents with myopia (*OR* = 3.822) had higher risks of myopia with no parental myopia, and these *ORs* were higher in Wuwei City than those of Gannan Prefecture. The regional differences of these *ORs* may correlate to the proportion of parental myopia in the two regions. The proportion of parental myopia was 49.4% among students in Wuwei City, while was only 21.5% in Gannan Prefecture. Research from Japan found that longer axial length (AL) or higher AL to the corneal radius of curvature (AL/CR) was significantly associated with parental myopia (24). While AL and AL/CR could be used as obtainable indicators for identifying subjects at high risk of developing premyopia and myopia in young preschool children (25). It has been suggested that students with a family history of myopia should be prioritized for intervention to prevent and manage myopia onset and progression from an early age.

The results of our study indicated the difference in gender and education levels of myopia. The risk of myopia was higher among girls than that of boys and increased with grade level, which was consistent with the findings of other studies (26–28). Possible reason, one aspect may involve the physiological differences in AL and corneal curvature (CR) among children of different genders and ages. As age progresses, AL tends to increase, and girls typically exhibit a deeper CR compared to boys (29). As discussed earlier (25), an increase in AL, deeper CR, and a combination of these changes in AL/CR were associated with myopia. However, this inference could be attributed to not performing revision under cycloplegia (24). Another possibility could be the influence of environmental factors.

In addition to genetics, the environment can also influence the development of myopia. Zhang et al. (30) found that genetic factors may play a role of 12.5%, while environmental factors may play a role of 87.5% in the formation of myopia, which suggests that environmental factors play an essential role in myopia. In the present study, we found that environmental factors, which included time spent on homework, incorrect reading and writing posture, mean time between continuous eye use, doing eye exercises, and average daily sleep were correlated with myopia.

The findings in this study indicated that the risks of myopia were higher among the students who spent less than 2 h compared to those with more than 2 h of homework. There may be three possible explanations for these findings. First, the homework burden increases with grades, which may lead to a reduction in exercise time. Previous studies had confirmed that exercise was a protective factor against myopia. Increasing outdoor time reduced the risk of myopia onset and myopic shifts, especially in non-myopic children, and daily outdoor time of 120 to 150 min at 5,000 lux/min or cumulative outdoor light intensity of 600,000 to 750,000 lux significantly reduced the incidence risk ratio of myopia by 15% ~ 24% (14, 31). Tracing the causes, the increasing outdoor physical activity time against myopia was mainly attributed to increasing vitamin D and dopamine levels (32–34). Girls were found to spend less time on outdoor physical activities compared to boys (35, 36). While physiological parameters are beyond control, it remains crucial to promote increased activity time to mitigate and manage myopia. This emphasizes the significance of engaging in outdoor activities as much as possible and ensuring exposure to sunlight, particularly for girls, as a preventive measure against myopia (37). Second, increasing homework burden leads to more frequent use of eyes and eye strain for children. As a result, our study showed that the risk of myopia increased significantly when studying for more than a quarter without taking a break while doing eye exercises twice a day or more was the protective factor for total participants and children in Gannan Prefecture. However, the protection role of doing eye exercises for myopia was only found in univariate analysis in Wuwei City. Eye exercises are a kind of eye health exercise based on the knowledge of traditional Chinese medicine. When doing eye exercises, students were asked to regularly press some acupoints of two eyes with thumbs for 10–15 s. These acupoints include Jingming point, Sizhukong point, Zhuzhu point, Tongziliao point, Yuyao point, Chengxi point, Sibai point, Yifeng point, and Yiming point (Table 6). Doing eye exercises may regulate the blood circulation and muscles of the eye and improve eye fatigue, so as to achieve the purpose of preventing and controlling myopia (38–40). Wang et al. (41) also found no evidence for an effect of eye exercises on change in vision. Lin et al. (42) found that only the seriousness of attitude toward performing the eye exercises of acupoints showed a protective effect toward myopia. The value of OR(95%CI) was 0.51(0.33–0.78) (42). Therefore, it is important for students to complete eye exercises with high quality rather than as a formality (43). Third, increasing homework burden also leads to less duration sleep time for children. There is controversy about the correlation between sleep and myopia. For example, in a cross-sectional study on Chinese children aged 6–18 years, univariate regression analysis showed that compared to sleep duration <8 h per day, children with sleep duration of 8–9 and > 9 h per day had lower rates of myopia. However, after adjusting for age, gender, parental myopia, outdoor time, and continuous near work duration without breaks, sleep duration was not associated with myopia (44). Xu et al. (45) found that nighttime sleep duration of <7 h/day (OR = 1.27, 95%CI: 1.17–1.38) was likely to be associated with increased risks of self-reported myopia after adjusting age, sex, grade, parental education level, family income, parental myopia, academic record, and academic workload. After reviewing 864 published articles, members of the American Academy of Sleep Medicine recommended children aged 6–12 years old and teenagers aged 13–18 years old should sleep 9 to 12 h and 8 to 10 h per 24 h on a regular basis to promote optimal health (46). According to the current study, sleep duration of <8 h per day was the dependent risk factor of myopia for total participants and children in Gannan Prefecture, while there was an independent association in Wuwei City. Ensuring 8 h of sleep may be conducive to the prevention and control of myopia. At present, the mechanism between sleep and myopia is not clear. The fundamental regulator of sleep depends on the circadian cycle in melatonin synthesis and release (47). It seemed reasonable that deprivation caused disorder of the circadian rhythm, delayed melatonin circadian rhythm phasing, and lower melatonin output, which led to myopia (48). Therefore, ensuring adequate sleep is beneficial for the eye health of children and teenagers.

**Table 6:** Location of the eye exercise point.

Name of acupoints	Location of acupoints
Jingming point	Above the medial canthus, depression of the medial wall of the orbit, the site of the supratrochlear nerve outflow
Sizhukong point	Above the lateral canthus, the lateral margin of the brows
Zhuzhu point	The location of the supraorbital notch
Tongziliao point	1.7 cm lateral to lateral canthus
Yuyao point	The intersection of the brows and the vertical line of throng the pupil
Chengxi point	Below the pupil, the location between the eyeball and the inferior orbital margin
Sibai point	The location of infraorbital foramen
Yifeng point	Posterior earlobe, anterior depression of lower end of mastoid
Yiming point	After the ear, the mastoid cavity between the mandibular angle and backward approximately 3.3 cm

Our study showed that incorrect reading and writing posture was correlated factors of myopia; the values of OR (95%CI) were 1.257 (1.108, 1.425), 1.278 (1.041, 1.569), and 1.204 (1.024, 1.416) among all participants, Gannan Prefecture and Wuwei City. Zhang et al. found that reading and writing with eyes more than one foot from books was a correlated factor for preventing myopia. The value of OR (95%CI) was 0.75 (0.68, 0.82) (49). A cross-sectional study from Xi’an, China, displayed that doing “one punch, one foot, one inch “(when reading and writing, 10 cm from the chest to the table, 33 cm from the eye to the book, and 3.3 cm from the tip of the pen to the finger) was also correlated with myopia. The value of OR (95%CI) was 0.81 (0.73–0.91) (50). Therefore, maintaining a correct reading and writing posture and keeping the eyes more than one foot away from books would be beneficial for preventing and controlling myopia. Our research further indicated that students who frequently or always received encouragement from their teachers regarding proper reading and writing posture had a lower prevalence of myopia (69.8%) compared to those who never or only occasionally received such encouragement (75.1%). Taking into account the fact that students spend a long time at school and are compliant with teachers, teachers should remind students to pay attention to good reading and writing posture and protect their eye health (51).

Our study has some limitations. First, the data on lifestyle, ocular behaviors, and environment were collected from a self-administered questionnaire, which may lead to recall bias. Second, refraction was determined by cycloplegic autorefraction in the international consensus for defining myopia. However, refraction was determined by non-cycloplegic autorefraction in the present study, which may result in an overestimation of myopia, while myopia was assessed by combining refractive correction and visual acuity to reduce this effect. Nevertheless, it is necessary to perform the refractive examination in cycloplegia to obtain more realistic data in further studies, which may be conducive to better prevention and control of myopia.

## 5 Conclusion

Our study confirms the role of both hereditary and environmental factors in the prevalence of myopia among students from two regions within Gansu Province, highlighting regional disparities. Students with parental myopia and girls should be the key population to prevent and control myopia by taking multiple intervention measures, including mainly homework reduction, outdoor activities in the presence of the sun, adequate lighting in reading-writing activities, reading and writing with correct posture, adequate sleep, doing eye exercises, controlling electronic device use, relaxing eyes after studying for some time, and frequent ophthalmologic evaluations.

## Data availability statement

The original contributions presented in the study are included in the article/supplementary material, further inquiries can be directed to the corresponding authors.

## Ethics statement

The studies involving humans were approved by the Ethics Committee of School of Basic Medical Sciences, Lanzhou University. The studies were conducted in accordance with the local legislation and institutional requirements. Written informed consent for participation in this study was provided by the participants' legal guardians/next of kin. Written informed consent was obtained from the minor(s)' legal guardian/next of kin for the publication of any potentially identifiable images or data included in this article.

## Author contributions

JW: Investigation, Methodology, Writing – original draft, Writing – review & editing. SL: Data curation, Investigation, Resources, Writing – review & editing. SH: Investigation, Writing – review & editing. YF: Investigation, Writing – review & editing. PL: Investigation, Writing – review & editing.
