# Corrigendum: Regional disparities in the prevalence and correlated factors of myopia in children and adolescents in Gansu, China

**DOI:** 10.3389/fmed.2024.1487929

**Published:** 2024-09-17

**Authors:** Jinyu Wang, Sheng Li, Shiqi He, Yali Feng, Pu Li

**Affiliations:** ^1^School of Public Health, Lanzhou University, Lanzhou, China; ^2^Department of Public Health, Lanzhou Second People's Hospital, Lanzhou, China; ^3^Department of Ophthalmology, Baiyin Second People's Hospital, Baiyin, China

**Keywords:** children and adolescents, myopia, influencing factors, regional disparity, prevention and control

In the published article, there was an error in Figures 1, 2. Figures 1, 2 had the text of the variable names incorrectly adjusted. These variables are now explained in the footnotes of the tables. The corrected Figures appear below.

**Figure 1:**
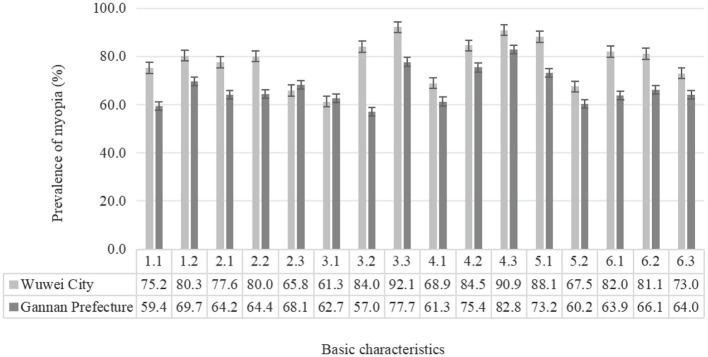
Prevalence of myopia of different basic characters students in the two regions (%). 1.1, Boys; 1.2, girls; 2.1, ethnic han; 2.2, Tibetan; 2.3, other ethnic; 3.1, elementary; 3.2, junior; 3.3, senior; 4.1, no parental myopia; 4.2, father or mother with myopia; 4.3, parents with myopia; 5.1, average daily sleep duration < 8 h; 5.2, average daily sleep duration ≥ 8 h; 6.1, daytime outdoor activity < 1; 6.2, daytime outdoor activity 1–2 h; 6.3, daytime outdoor activity ≥ 2 h.

**Figure 2:**
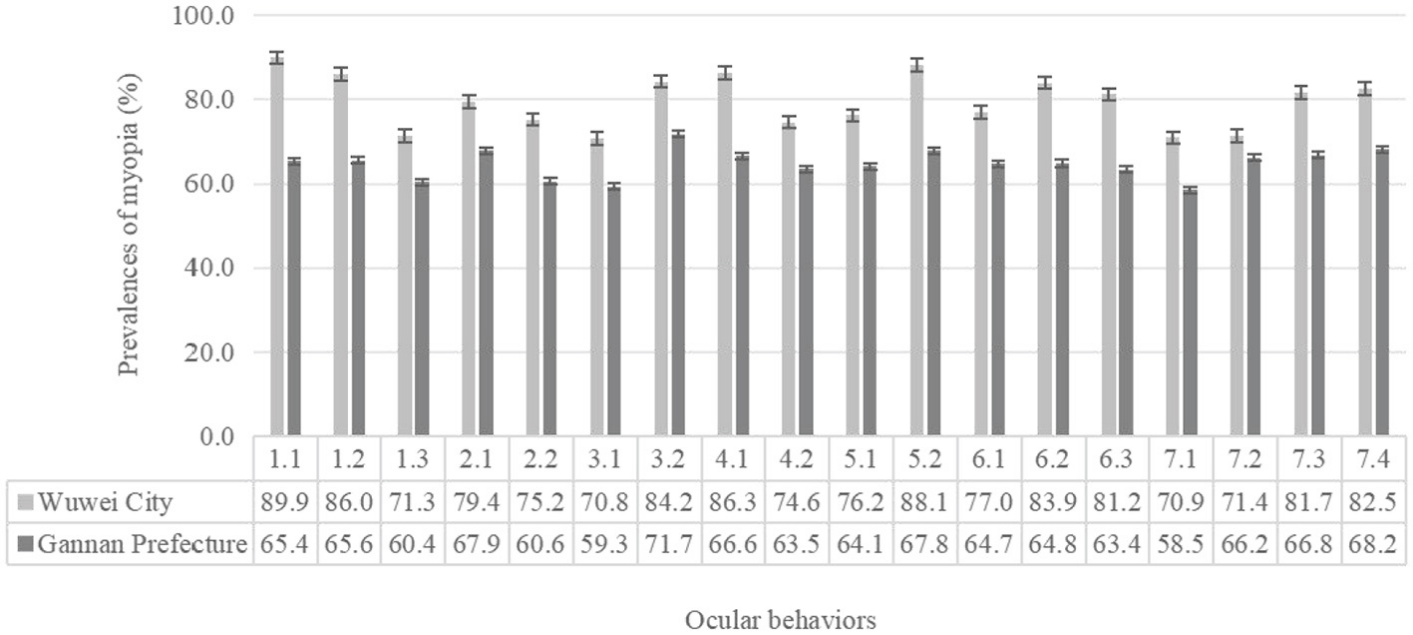
Prevalence of myopia of different ocular behaviors students in the two regions (%). 1.1, doing eye exercises 0 time/d; 1.2, doing eye exercises 1 time/d; 1.3, doing eye exercises ≥ 2 times/d; 2.1, recess in the teaching building; 2.2, recess outdoor; 3.1, daily time spend doing homework < 2 h; 3.2, daily time spend doing homework ≥ 2 h; 4.1, distance between eyes book less than one foot; 4.2, distance between eyes book more than one foot; 5.1, reading occasionally while reclined or lying down; 5.2, reading always while reclined or lying down; 6.1, daily time spend using computer < 1 h; 6.2, daily time spend using computer 1–2 h; 6.3, daily time spend using computer ≥ 2 h; 7.1, mean time continuous eye use < 0.25 h; 7.2, mean time continuous eye use 0.25–0.5 h; 7.3, mean time continuous eye use 0.5–1 h; 7.4, mean time continuous eye use ≥ 1 h.

The authors apologize for this error and state that this does not change the scientific conclusions of the article in any way. The original article has been updated.

